# Relational Approaches in Patient-Oriented Research During the COVID-19 Pandemic

**DOI:** 10.1093/geroni/igab046.1507

**Published:** 2021-12-17

**Authors:** Lillian Hung

**Affiliations:** University of British Columbia, Vancouver, British Columbia, Canada

## Abstract

The COVID-19 pandemic has exposed the fragile state of patient involvement in research. The involvement of the most vulnerable population (older people with dementia) in research was even more challenging. This presentation outlines challenges my research team encountered in patient-led projects (older people with dementia) and describes how we found creative strategies to set up and complete research during the time of pandemic. I will describe how the team applied collaborative participatory principles to engage a team with diverse backgrounds in the lockdown time to maintain research progress. Patient partners in my research team actively led recruitment, research planning and decision-making, team analysis and knowledge exchange. University students in our research team helped to make technology easy to use for our patient partners. The friendly, flexible and accessible exchange between students and patient partners reinforced the importance of a respectful relational approach in patient-oriented research.

